# Hierarchical Microstructure of Laser Powder Bed Fusion Produced Face-Centered-Cubic-Structured Equiatomic CrFeNiMn Multicomponent Alloy

**DOI:** 10.3390/ma13204498

**Published:** 2020-10-11

**Authors:** Xuan Yang, Yanling Ge, Joonas Lehtonen, Simo-Pekka Hannula

**Affiliations:** Department of Chemistry and Materials Science, Aalto University School of Chemical Engineering, P.O. Box 16100, FI-00076 Espoo, Finland; yanling.ge@aalto.fi (Y.G.); joonas.m.lehtonen@aalto.fi (J.L.); simo-pekka.hannula@aalto.fi (S.-P.H.)

**Keywords:** high-entropy alloys, laser powder bed fusion, selective laser melting, solidification microstructure

## Abstract

A cobalt-free equiatomic CrFeNiMn multicomponent alloy was fabricated from gas-atomized powder using laser powder bed fusion (L-PBF), also known as selective laser melting (SLM). The as-built specimens had a single face-centered cubic (FCC) structure, relative density of 98%, and hardness up to 248 HV_0.5_ for both the scanning speeds applied. In this work, we report the hierarchical microstructural features observed in the as-built specimens. These are comprised of melt pools, grains, cell structures including dendritic cells, elongated cells, equiaxed cells (~500 nm), and sub-cells (150–300 nm). The cell and sub-cell walls are composed of a notably high density of dislocations. In addition, segregation of Mn and Ni was detected at the cell walls, but only occasionally at the sub-cell walls. SLM exhibits the capability to produce FCC-structured equiatomic CrFeNiMn multicomponent alloy with the refined and hierarchical microstructure.

## 1. Introduction

The traditional alloys invented and developed through history are produced by mixing two or more elements, one of which is a metal at least domaining the composition, known as the base or primary metal. After mixing metals and other elements together, important and desirable properties can be achieved. In recent years, the concept of manufacturing alloys with more than one or two principal elements has been considered to be promising to introduce innovations in alloys [1,2]. The multicomponent alloys, generally known as high-entropy alloys (HEAs), provide excellent properties beyond traditional metal alloys, including high strength [3,4], enhanced plasticity [5], oxidation resistance [6], wear resistance [7], and hydrogen storage ability [8]. Among the HEAs, the representative CoCrFeNiMn alloy has been the most widely studied [1,9]. However, for some specific applications, such as in the nuclear field, cobalt-free alloys are preferred because of the high cost and activation of cobalt-containing alloys. All kinds of materials have recently been considered promising as the structural materials of next-generation nuclear reactors, including austenitic stainless steels and nickel-base superalloys [10]. In this research, we introduce the probability of using multicomponent alloys, by removing the cobalt alloying element and producing an equiatomic CrFeNiMn multicomponent alloy.

Research on equiatomic CrFeNiMn alloy consisting of a single face-centered cubic (FCC) structure has rarely been reported before. In the quaternary Cr-Fe-Ni-Mn system, Cr is considered a stabilizer for body-centered cubic (BCC) structures, and according to former research [11], its content should be kept below 18 wt% to prevent the FCC phase from being destabilized by the presence of Mn under the homogenization condition. Based on this, Wu et al. [12] developed the nonequiatomic FeNiMnCr_18_ alloy (Cr content was 18 at%, and the remaining elements were equiatomic) composed of a single FCC phase by arc melting, and found that the strength and ductility of the alloy were comparable with those of CoCrFeNiMn alloy. Further research of this cobalt-free alloy [13] confirmed its superior radiation resistance ability compared to conventional austenitic stainless steels. Nevertheless, former research [14] has observed that the single-FCC-structured equiatomic CrFeNiMn alloy cannot be produced using traditional manufacturing methods. In addition to that, as multicomponent alloys possess a complex system, it would require higher control ability and cost to produce them traditionally.

Laser powder bed fusion (L-PBF), commonly known as selective laser melting (SLM), is one of the compelling additive manufacturing (AM) technologies developed in the past two decades. It utilizes a laser beam to consolidate metallic powder layer by layer into bulk material, especially with complex geometrical features, and without the need for post-processing [15]. During the SLM process, the powder is fully melted, and a new rapidly solidified microstructure is produced, contributing to the formation of novel compositions and refinement of microstructure [16]. Several studies have explored the printability of different multicomponent alloys, including FeCoCrNi [17], CoCrFeNiMn [18], AlCrCuFeNi [19], and AlxCoCrFeNi [20]. Brif et al. [17] were the first who successfully produced FeCoCrNi alloy consisting of single-phase solid solution using SLM, and found that its high strength and ductility were comparable to stainless steels due to the refined structure induced by SLM. Zhu et al. [18] studied the hierarchical microstructure of the SLM CoCrFeNiMn alloy, including melt pools, columnar grains, cellular structures, and dislocations, which contribute to the superior combination of high strength and ductility compared to those fabricated by conventional methods. Yet, the majority of multicomponent alloys is still manufactured by traditional methods; especially, CrFeNiMn alloy fabricated by SLM has hardly been reported. Refining the microstructure through the SLM process offers a promising strategy that could promote exciting new developments of multicomponent alloys.

The feasibility of fabricating single-FCC-structured equiatomic CrFeNiMn alloy using SLM has been studied in our preliminary work, while little is known about its microstructural features. The purpose of this research is to perform detailed microscopic studies on the FCC-structured CrFeNiMn alloy produced by SLM applying two sets of scanning speeds. The effects of the SLM process on the phase constitution, chemical composition, and microstructure are characterized, and the formation mechanism of the microstructure is discussed. 

## 2. Materials and Methods 

Gas-atomized CrFeNiMn alloy powder with a particle class size between 20 and 45 μm was selected, as gas atomization provides the fine spherical powder with high density that is suited to the SLM process [21]. The powder constituted a near-single FCC phase with a small amount of BCC phase, as described in the earlier publication [22]. 

An SLM-50 machine (Realizer GmbH, Paderborn, Germany) equipped with a laser source of 120 W maximum power output and 50 μm-diameter spot size was employed. The initial SLM parameters were based on the work of Liu et al. [23] on the SLM of 316L stainless steel. They achieved a density of 98% and above with 50 W laser power, 100–300 mm/s scan speed, 80 μm hatch spacing, and 48 μm-diameter spot size. The preliminary experiments yielded two series of samples. One set of samples had a low density while the second series of samples turned out to have acceptable density. Out of this latter set, two samples were selected for further study in the present paper. The detailed study of the effect of SLM parameters on the properties of the samples will be reported soon. The following parameters, i.e., laser power *P* of 70 W, hatch spacing *h* of 98 μm, and layer thickness *t* of 25 μm, were adopted. Two specimens, SLM-1 and SLM-2, were produced corresponding to a scanning speed *v* of 200 and 150 mm/s, and two energy density values *E* of 142.86 and 190.48 J/mm^3^, respectively. The process was performed under a protective Ar atmosphere, and the oxygen level was maintained below 0.2%. The island scanning pattern with islands of 5 mm × 5 mm was used [24], and each specimen had a size of 20 mm × 20 mm × 3 mm. The layer rotation used was 90°.

All the characterizations of as-built specimens in this work were carried out on the XY-plane. The phase constituents of the powder and SLM CrFeNiMn alloy specimens were analyzed by X-ray diffraction (XRD, X’ Pert PRO MPD, PANalytical, Almelo, The Netherlands) applying Co-Kα (1.7903 Å) radiation under 40 mV voltage and 40 mA current with a step size of 0.0131°. The surface morphology and chemical composition of SLM specimens were studied by a focused-ion-beam scanning electron microscope (FIB-SEM, JIB-4700F, JEOL, Tokyo, Japan) equipped with an energy-dispersive X-ray spectrometer (EDS). For each average chemical composition, 40 points from different areas of each specimen were measured. The chemical composition of the powder was measured by a wavelength-dispersive X-ray fluorescence spectrometer (WDXRF, Axios mAX 3 kW, PANalytical, Almelo, The Netherlands). Transmission electron microscope (TEM) samples were prepared of slices cut from the SLM specimen. They were ground with fine SiC paper from P120 to P1200 to an approximate thickness of 100 μm. Then, 3 mm-diameter discs were punched from the slices. Discs were electro-polished in a twin jet polisher (TenuPol-5, Struers, Ballerup, Denmark) with 13 v% HNO_3_-ethanol solution at −23 °C under 20.5 V. The TEM studies were carried out with a JEM-2800 (JEOL, Tokyo, Japan) equipped with EDS. The theoretical density was calculated to be 7.80 g/cm^3^, and the relative density was determined through the Archimedes method. The average cell size, relative cell size distribution, and volume-weighted average cell size were estimated by a MATLAB script (MATLAB R2020a version 9.8) created by Lehto et al. [25], and at least 200 cells were analyzed for each specimen. The cross-sectional area proportion of the cell walls (% of total area) was estimated using the ImageJ program (v. 1.52p). The hardness was examined by the low-force Vickers hardness tester (Nexus 4303, Innovatest, Maastricht, The Netherlands) according to ISO 6507-1:2018 [26], at a force of 4.903 N and a dwell time of 10 s. Nine measurements for each specimen were carried out.

## 3. Results and Discussion

### 3.1. Microstructure of Gas-Atomized Powder

**Figure 1:**
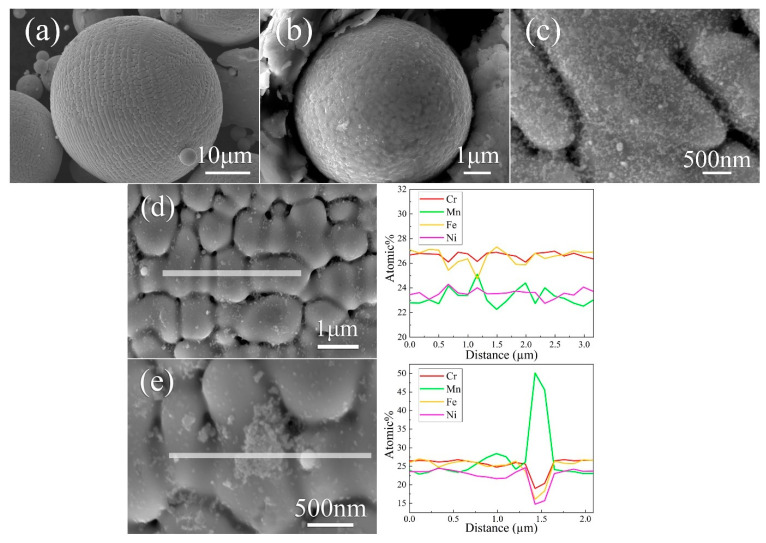
Surface morphology and EDS line scan on the surface of CrFeNiMn powder. The SE micrographs of (**a**) large particle; (**b**) small particle; (**c**) debris on a random powder surface. The EDS line scan (**d**) across cell structures and (**e**) across one nano-inclusion and -debris.

The secondary electron (SE) images reveal the gas-atomized powder particles possessing two different surface morphologies depending on the size (Figure 1a,b). On the surface of the larger particles (Figure 1a), columnar cell structures are distributed in several dendritic areas with some elongated cells up to 10 μm long. On the surface of the smaller particles (Figure 1b), evenly distributed equiaxed cells with a size < 1 μm are found. Moreover, nanoscale debris is found to disperse nonuniformly on the surface of the particles (Figure 1c). EDS linear scanning results in Figure 1d,e illustrate the variation in four elements on the particle surface. As delineated in Figure 1d, Mn along with Ni is slightly enriched at the cell walls, the content of Mn is in the interval from 22.3 to 25.1 at%, and Mn varies more notably than that of Ni in the range of 22.7–24.3 at% from the cell walls to the interior of cells. Fe content decreases to 24.7 at% when encountering the cell walls and increases up to 27.3 at% toward the middle of the cells. Cr has a similar effect as Fe with a small fluctuation, and it remains relatively stable in the range of 26.1–26.9 at%. Figure 1e demonstrates that Mn dominates the chemical composition of the nano-inclusion with a content up to 50.1 at%, and nano-debris with a content up to 28.4 at%. Cr and Fe are depleted in the nano-debris, nano-inclusion, and cell wall, while Ni is depleted in the debris and inclusion but enriched in the cell wall.

### 3.2. Density, Chemical Composition, and Phase Constituent

Table 1 summarizes the relative density and chemical composition of the powder and SLM specimens. A relative density of 98.0% ± 0.1% is achieved for SLM-1 with a scanning speed of 200 mm/s. Decreasing the scanning speed to 150 mm/s in SLM-2 makes little diversity, as the relative density is of 97.9% ± 0.2%. One of the origins of porosity could be attributed to the satellites observed in Figure 1a. It was studied previously by Ali et al. [27] that satellites in powder could lead to incomplete melting in the powder layer, and trap gases in those regions; as a consequence, a high amount and large pores were found in the SLM specimens. It can be seen from Table 1 that the compositions of the SLM specimens change based on the process parameters used in the SLM process. Particularly when the scanning speed is decreased, resulting in the rise in energy density, the Mn content in the specimen is decreased. Compared to the original composition of Mn in powder, SLM-2 experiences more loss in the SLM process, while SLM-1 retains a similar content in the SLM process. The decrease in Mn content in SLM specimens is attributed to the vaporization of Mn during the laser melting process. When a high-power laser beam focuses on a relatively small area and fuses powders, the temperature rises, and the volatile elements evaporate. Similar effects have been observed with other Mn-containing complex alloys during the SLM process [28]. 

**Figure 2:**
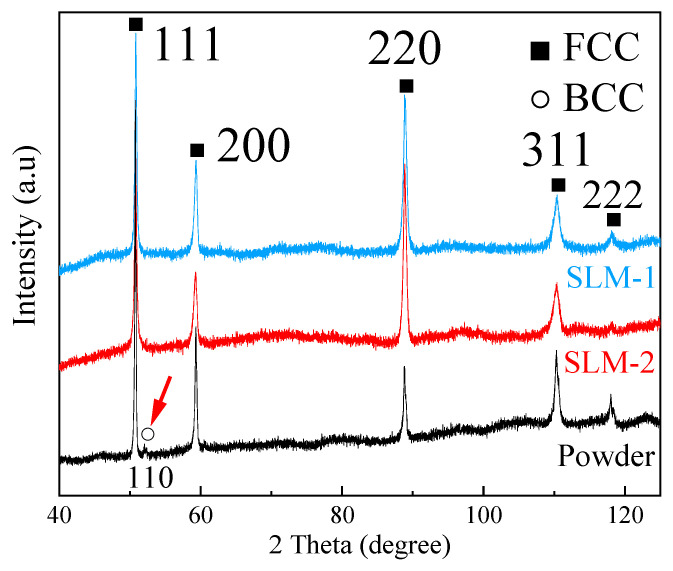
XRD patterns of CrFeNiMn powder and SLM CrFeNiMn alloy.

Figure 2 illustrates the XRD patterns of powder and SLM specimens. The high relative intensity of the 220 peak indicates that the preferred crystallographic orientation was developed in SLM specimens. In the starting powder, the presence of the BCC phase is confirmed, as a small BCC 110 peak is visible, indicated with a red arrow in Figure 2. Nevertheless, after SLM, there is no detectable BCC phase in specimens. 

In our former observation [22], an increasing amount of BCC phase was detected with decreasing powder size. In solidification, the probability of potent heterogeneous nucleants in liquid is proportional to the liquid volume. As the smallest droplets are least likely to contain a nucleant, they tend to reach the greatest supercooling prior to the nucleation of the crystal, compared to large droplets. It indicates that the small liquid droplets that solidify into small powder particles experience a greater supercooling than the large liquid droplets that solidify into large powder particles. Moreover, the BCC phase tends to nucleate when supercooling is large. This observation agrees with that of Kelly et al. [29], whom discovered that the nucleation of the BCC phase in an Fe-Cr-Ni stainless steel became possible under high liquid supercooling. Raghavan [11] claimed that a CrFeNiMn equiatomic alloy will be completely FCC only if the Cr content were reduced to 18 wt%. Formation of the BCC phase in the small powder particles is thus proposed to result from the isolation of heterogeneous catalysts. During the SLM process, however, heterogeneous nucleants are readily available in the large melt volume and on the already existing solid surfaces. Therefore, the degree of supercooling in SLM is comparable to powder particles of large size. Heterogeneous nucleation on existing solid FCC phase surfaces may also result in the epitaxial growth of solid. This is evidenced by the high intensity of 220 peaks in the XRD diffractograms (Figure 2). Accordingly, the single FCC phase structure is observed in SLM specimens. This result demonstrates that the solidification mode in the SLM process favors the formation of a single FCC phase structure in equiatomic CrFeNiMn alloy, which makes SLM ideal to fabricating this cobalt-free multicomponent alloy.

### 3.3. Microstructure of SLM CrFeNiMn Alloy

**Figure 3:**
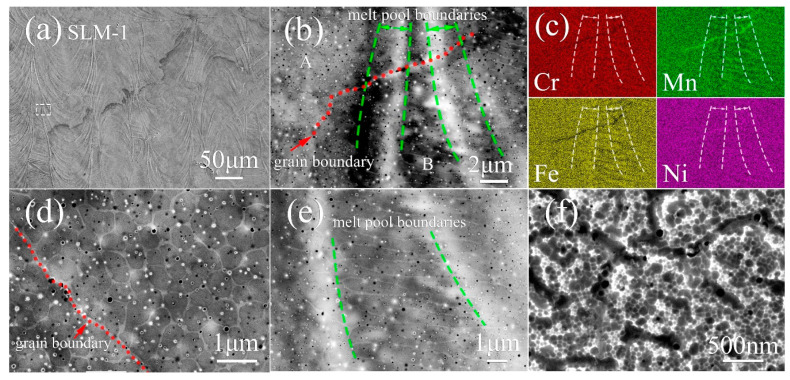
SEM observation on the XY-plane of SLM-1 CrFeNiMn alloy. The SE images of (**a**) melt pool assemblages; (**b**) detailed microstructure with (**c**) the corresponding quantitative SEM-EDS maps of elemental distribution; (**d**) equiaxed cells; (**e**) elongated cells; (**f**) ultrafine sub-cells. The micrographs in (**d**,**e**) present the typical magnified morphology of area A and B in (**b**), respectively.

**Figure 4:**
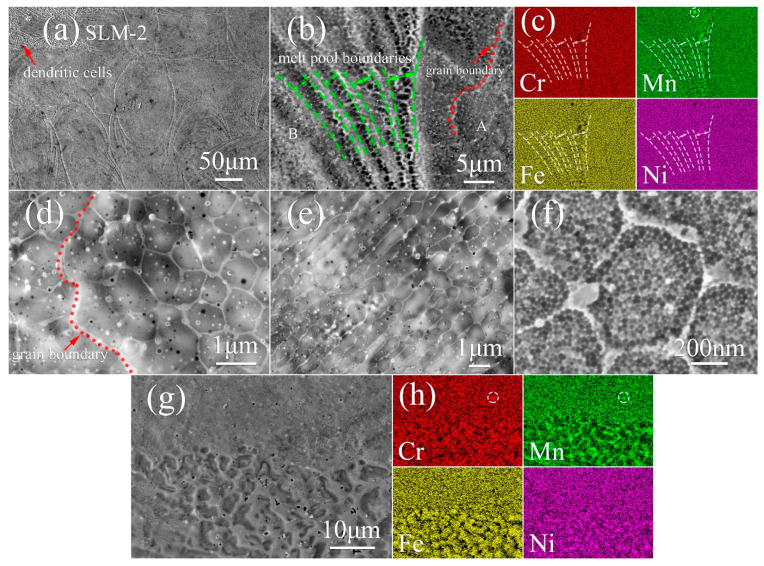
SEM observation on the XY-plane of SLM-2 CrFeNiMn alloy. The SE images of (**a**) melt pool tracks and dendrites; (**b**) detailed microstructure with (**c**) the corresponding quantitative SEM-EDS maps of elemental distribution; (**d**) equiaxed cells; (**e**) elongated cells; (**f**) ultrafine sub-cells; and (**g**) magnified dendritic cells with (**h**) the corresponding quantitative SEM-EDS maps of elemental distribution. The micrographs in (**d**,**e**) present the typical magnified morphology of area A and B in (**b**), respectively.

SLM specimens are found to exhibit a hierarchical microstructure. Figure 3 and Figure 4 display the SE micrographs of the chemically etched XY-plane of SLM-1 and SLM-2, respectively. The XY-plane refers to the layer that is produced perpendicularly to the building direction in the additive manufacturing process. In SLM-1, melt pools are observed in Figure 3a. Figure 3b presents the magnified rectangular area depicted in Figure 3a, and discloses the typical microstructural features, namely, melt pool boundaries, grain boundaries, equiaxed cells (area A), elongated cells (area B), and nano-inclusions. Equiaxed cells (magnified in Figure 3d) are found relatively far from the melt pool boundaries, while elongated cells (magnified in Figure 3e) are distributed close to the melt pool boundaries and appear approximately perpendicular to the boundaries, which are formed in the beginning of solidification due to epitaxial growth. The formation of these two types of cells is ascribed to the different temperature gradient and solidification velocity at the solid–liquid interface in different locations of the melt pool, especially at the edge or the bottom [30]. Nano-inclusions are more likely to be embedded at cell walls (Figure 3d,e). The elemental distribution maps of Figure 3b presented in Figure 3c show a slight enrichment of Mn and Ni at the grain boundaries and cell walls, where Cr and Fe are depleted. By contrast, Fe and Cr are enriched around melt pool boundaries. Furthermore, in most of the areas of SLM-1, ultrafine sub-cells are found inside the general cells (Figure 3f).

In SLM-2, apart from the wider melt pools, dendritic cells are observed along melt pool boundaries, indicated with a red arrow in Figure 4a. Generally, dendrites are found to grow along the deposition direction, which is opposite to the direction of heat flux [31]. When employing the island scanning strategy, two adjacent and parallel melt pool tracks always overlap with each other, and the overlap area of melt pool tracks is always re-melted by the next scan. The cooling of melt pools initiates from the substrate or previously solidified parts, from which dendritic cells grow epitaxially. The elemental maps (Figure 4g,h) show that dendritic cell walls possess a similar enrichment of Mn and Ni, and depletion of Cr and Fe as the other cell walls, but they are much wider than the general cell walls such as in Figure 4d,e. A similar microstructure is observed in SLM-2 (Figure 4b) along with the segregation of Mn and Ni into cell walls and grain boundaries (Figure 4c). The circled particle in Figure 4c indicates the presence of Mn-enriched nano-inclusions, like those observed in powder (Figure 1e). Area A and B correspond to equiaxed cells (magnified in Figure 4d) and elongated cells (magnified in Figure 4e), respectively. Both types of cells are located in the identical areas of SLM-1. In addition, ultrafine sub-cells are also identified in SLM-2 (Figure 4f). The particle circled in Figure 4h displays the existence of Mn-Cr-enriched nano-inclusion. 

**Figure 5:**
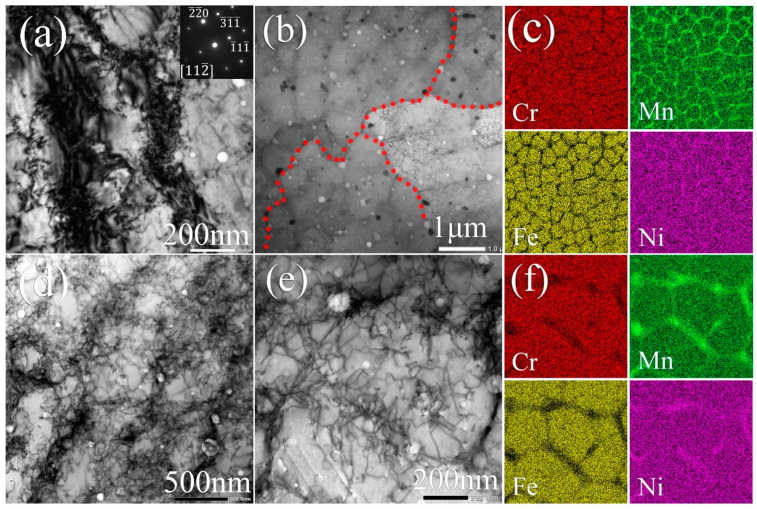
TEM observation on the XY-plane of SLM-2 CrFeNiMn alloy. (**a**) BF TEM image of equiaxed cells with the corresponding selected area electron diffraction (SAED) pattern. BF STEM image of (**b**) equiaxed cells with (**c**) corresponding quantitative EDS maps; (**d**) detailed equiaxed cells and dislocation networks and (**e**) magnified equiaxed cells with (**f**) corresponding quantitative EDS maps of elemental distribution in the sub-cell structure.

Further TEM investigation on the XY-plane of SLM-2 is displayed in Figure 5. The bright-field (BF) TEM image in Figure 5a reveals a great density of dislocations concentrated mainly along the cell walls and, to some extent, within the cells. The BF-STEM (bright field scanning transmission electron microscopy) image in Figure 5b presents the dislocations to tangle into networks forming cell structures inside grains. The element maps of the area shown in Figure 5b are presented in Figure 5c. They confirm the segregation of Mn and Ni into cell walls and grain boundaries. The Mn map reveals a large amount of Mn-enriched inclusions that are almost all embedded at cell walls. Similarly, the Cr map shows that the majority of Cr-enriched inclusions are located near the cell walls, with a few inclusions located within cells. Moreover, it is found that the sub-cells with a size in the range of 150–300 nm are formed by dislocations networks in the interior of cells (Figure 5d). Inside a single cell, the enrichment of Mn occurs mainly at sub-cell walls where a higher density of dislocations is simultaneously found, while some part of the sub-cell walls discloses dislocation networks without segregation (Figure 5e,f). The temperature gradient and time-varying processing temperatures in SLM [32] are responsible for the formation of the dislocations, owing to them inducing internal stresses and localized strains. More than that, all the microstructures observed in SLM specimens result from different temperature gradients. Different microstructures would etch at different rates during the electrochemical polishing, and be revealed as hierarchical features. 

In the incipient stage of solidification, the elements with a higher melting point tend to enrich in the solid while the lower-melting elements, i.e., Mn and Ni, tend to enrich to the melt at the solid–liquid interface [33]. As solidification proceeds, proportions of regions protrude into the liquid faster than the rest of the interface, leading to cellular structure. Thus, the concentration of lower-melting elements is higher around the protuberances, which explains the enrichment of Mn and Ni in grain boundaries and cell walls, and the depletion of them around melt pool boundaries, where Cr and Fe are enriched. The selection of proper processing parameters is crucial for preservation of the desired chemical composition, especially when elements with high vapor pressure such as manganese are present. Vaporization of manganese occurs during the melting process. However, some vapors may recondense in the form of spherical nano-inclusions embedded in cell walls (Figure 1d,e), and it is likely that some vapors recondense as nano-debris on the surface of powder in gas atomization as well (Figure 1c). In relation to Mn-Cr-enriched nano-inclusions (Figure 4h), due to Mn and Cr possessing a higher affinity to oxygen than Fe and Ni [34], Mn and Cr consequently react with residual oxygen in the chamber of the SLM unit. These nano-inclusions are found to retard the migration of dislocations, and introduce an enhanced strength [35]. 

### 3.4. Hardness

The solidified microstructure is dependent on the combination of temperature gradient *G* and growth rate *R* [36]. The *G*/*R* ratio governs the microstructural morphology, which is observed to be cellular in this research. The cooling rate *GR* determines the scale of microstructure, and a finer microstructure would be produced by increasing the cooling rate. Table 2 shows the comparison of equiaxed cell size parameters and the Vickers hardness of SLM specimens. The cell size parameters are found to be different when applying different scanning speeds. SLM-1, which has experienced a higher scanning speed resulting in higher cooling rate, discloses an average cell size of 390 nm, relative cell size dispersion of 1.85, volume-weighted average cell size of 450 nm, and cell wall cross-sectional area proportion of 14.70%. Each parameter relating to equiaxed cells of SLM-1 is smaller than that of SLM-2, which experienced a lower cooling rate. Correspondingly, SLM-1 shows a higher hardness up to 248 ± 8 HV_0.5_. It suggests that finer and more uniform cell structures with less segregation are produced under a higher scanning speed, which strongly contributes to better mechanical properties. Furthermore, the Vickers hardness of both specimens is notably higher than that of conventionally fabricated CoCrFeMnNi alloy (~160 HV_1_) [37]. The differences may be ascribed to the combination of fine cell structure, high density of dislocations, nano-inclusions, and residual stresses due to the SLM process. Former research [18,38,39] reported that the pre-existing dislocation networks induced by the SLM process provide substantially enhanced strength and ductility at the same time. The observation demonstrates the fact that by controlling the size of cell structures and chemical composition in conjunction with inducing dense dislocation networks and nano-inclusions, the SLM process can be utilized to reach the desired mechanical properties through optimizing the processing parameters.

## 4. Conclusions

To summarize, the equiatomic CrFeNiMn alloy was produced by the means of SLM. The formation of the FCC phase was favored, resulting in a single FCC structure in as-built SLM CrFeNiMn specimens. The hierarchical microstructural features were revealed on the xy-planes of SLM specimens, i.e., melt pools, grains, dendritic cells, elongated cells, equiaxed cells, and sub-cells (150–300 nm). The increase in scanning speed induced the formation of finer and more uniform cell structures with less segregation and higher hardness (248 ± 8 HV_0.5_). Mn and Ni elements segregated at grain boundaries and cell walls, and Cr and Fe were slightly enriched around melt pool boundaries. The cell walls constituted a high density of dislocations in conjunction with the inhomogeneous distribution of Mn and Ni alloying elements. The sub-cell walls consisted of dislocations associated within the cells, but only a part of the walls was enriched with Mn and Ni. SLM possesses a good controllability in refining microstructure, and produces hierarchical features resulting in improved mechanical properties unlikely to be produced by conventional methods.

## Figures and Tables

**Table 1:** Relative density and chemical composition (in at%) of selective laser melting (SLM) CrFeNiMn alloy specimens. Chemical composition (in at%) of CrFeNiMn powder.

Specimen	Cr	Mn	Fe	Ni	Relative Density
Powder	26.9	25.1	24.5	23.5	/
SLM-1	25.5	24.9	24.8	24.8	98.0% ± 0.1%
SLM-2	25.8	24.0	25.3	24.9	97.9% ± 0.2%

**Table 2:** Average cell size, relative cell size dispersion, volume-weighted average cell size of equiaxed cells, and Vickers hardness of SLM CrFeNiMn alloy specimens.

	Scanning Speed mm/s	Average Cell Size/nm	Relative Cell Size Dispersion	Volume-Weighted Average Cell Size/nm	Cell Wall Proportion	Vickers Hardness/HV_0.5_
SLM-1	200	390	1.85	450	14.70%	248 ± 8
SLM-2	150	520	2.16	680	15.17%	221 ± 14

## References

[1] Cantor B., Chang I.T.H., Knight P., Vincent A.J.B. (2004). Microstructural development in equiatomic multicomponent alloys. Mater. Sci. Eng..

[2] Huang P.-K., Yeh J.-W., Shun T.-T., Chen S.-K. (2004). Multi-Principal-Element Alloys with Improved Oxidation and Wear Resistance for Thermal Spray Coating. Adv. Eng. Mater..

[3] Otto F., Dlouhý A., Somsen C., Bei H., Eggeler G., George E.P. (2013). The influences of temperature and microstructure on the tensile properties of a CoCrFeMnNi high-entropy alloy. Acta Mater..

[4] LaRosa C.R., Shih M., Varvenne C., Ghazisaeidi M. (2019). Solid solution strengthening theories of high-entropy alloys. Mater. Charact..

[5] Fu Z., MacDonald B.E., Zhang D., Wu B., Chen W., Ivanisenko J., Hahn H., Lavernia E.J. (2018). Fcc nanostructured TiFeCoNi alloy with multi-scale grains and enhanced plasticity. Scr. Mater..

[6] Wang W., Wang J., Sun Z., Li J., Li L., Song X., Wen X., Xie L., Yang X. (2020). Effect of Mo and aging temperature on corrosion behavior of (CoCrFeNi)100-Mo high-entropy alloys. J. Alloys Compd..

[7] Löbel M., Lindner T., Lampke T. (2018). Enhanced Wear Behaviour of Spark Plasma Sintered AlCoCrFeNiTi High-Entropy Alloy Composites. Materials.

[8] Sahlberg M., Karlsson D., Zlotea C., Jansson U. (2016). Superior hydrogen storage in high entropy alloys. Sci. Rep..

[9] Gao M.C., Yeh J.-W., Liaw P.K., Zhang Y. (2016). High-Entropy Alloys.

[10] Murty K.L., Charit I. (2008). Structural materials for Gen-IV nuclear reactors: Challenges and opportunities. J. Nucl. Mater..

[11] Raghavan V. (1995). Effect of manganese on the stability of austenite in Fe-Cr-Ni alloys. Metall. Mater. Trans. A.

[12] Wu Z., Bei H. (2015). Microstructures and mechanical properties of compositionally complex Co-free FeNiMnCr_18_ FCC solid solution alloy. Mater. Sci. Eng..

[13] Kumar N.A.P.K., Li C., Leonard K.J., Bei H., Zinkle S.J. (2016). Microstructural stability and mechanical behavior of FeNiMnCr high entropy alloy under ion irradiation. Acta Mater..

[14] Wu Z., Bei H., Otto F., Pharr G.M., George E.P. (2014). Recovery, recrystallization, grain growth and phase stability of a family of FCC-structured multi-component equiatomic solid solution alloys. Intermetallics.

[15] Hooreweder B.V., Moens D., Boonen R., Kruth J.-P., Sas P. (2012). Analysis of Fracture Toughness and Crack Propagation of Ti6Al4V Produced by Selective Laser Melting. Adv. Eng. Mater..

[16] Lavernia E.J., Srivatsan T.S. (2010). The rapid solidification processing of materials: Science, principles, technology, advances, and applications. J. Mater. Sci..

[17] Brif Y., Thomas M., Todd I. (2015). The use of high-entropy alloys in additive manufacturing. Scr. Mater..

[18] Zhu Z.G., Nguyen Q.B., Ng F.L., An X.H., Liao X.Z., Liaw P.K., Nai S.M.L., Wei J. (2018). Hierarchical microstructure and strengthening mechanisms of a CoCrFeNiMn high entropy alloy additively manufactured by selective laser melting. Scr. Mater..

[19] Luo S., Gao P., Yu H., Yang J., Wang Z., Zeng X. (2019). Selective laser melting of an equiatomic AlCrCuFeNi high-entropy alloy: Processability, non-equilibrium microstructure and mechanical behavior. J. Alloys Compd..

[20] Joseph J., Jarvis T., Wu X., Stanford N., Hodgson P., Fabijanic D.M. (2015). Comparative study of the microstructures and mechanical properties of direct laser fabricated and arc-melted Al_x_CoCrFeNi high entropy alloys. Mater. Sci. Eng..

[21] Fritsching U., Uhlenwinkel V., Kondoh K. (2012). Hybrid Gas Atomization for Powder Production. Powder Metallurgy.

[22] Lehtonen J., Ge Y., Ciftci N., Heczko O., Uhlenwinkel V., Hannula S.-P. (2020). Phase structures of gas atomized equiatomic CrFeNiMn high entropy alloy powder. J. Alloys Compd..

[23] Liu B., Wildman R., Tuck C., Ashcroft I., Hague R. Investigation the effect of particle size distribution on processing parameters optimisation in selective laser melting process. Proceedings of the International solid freeform fabrication symposium: An additive manufacturing conference.

[24] Kamath C., El-dasher B., Gallegos G.F., King W.E. (2014). Density of additively-manufactured, 316L SS parts using laser powder-bed fusion at powers up to 400W. Int. J. Adv. Manuf. Technol..

[25] Lehto P., Remes H., Saukkonen T., Hänninen H., Romanoff J. (2014). Influence of grain size distribution on the Hall–Petch relationship of welded structural steel. Mater. Sci. Eng..

[26] (2018). Metallic Materials—Vickers Hardness Test—Part 1: Test Method.

[27] Ali U., Esmaeilizadeh R., Ahmed F., Sarker D., Muhammad W., Keshavarzkermani A., Mahmoodkhani Y., Marzbanrad E., Toyserkani E. (2019). Identification and characterization of spatter particles and their effect on surface roughness, density and mechanical response of 17-4 PH stainless steel laser powder-bed fusion parts. Mater. Sci. Eng..

[28] Nilsén F., Ituarte I.F., Salmi M., Partanen J., Hannula S.-P. (2019). Effect of process parameters on non-modulated Ni-Mn-Ga alloy manufactured using powder bed fusion. Addit. Manuf..

[29] Kelly T.F., Cohen M., Sande J.B. (1984). Rapid solidification of a droplet-processed stainless steel. Metall. Trans. A.

[30] Tan J.H.K., Sing S.L., Yeong W.Y. (2020). Microstructure modelling for metallic additive manufacturing: A review. Virtual. Phys. Prototyp..

[31] Bi G., Sun C.-N., Chen H., Ng F.L., Ma C.C.K. (2014). Microstructure and tensile properties of superalloy IN100 fabricated by micro-laser aided additive manufacturing. Mater. Des..

[32] Kruth J.P., Froyen L., Van Vaerenbergh J., Mercelis P., Rombouts M., Lauwers B. (2004). Selective laser melting of iron-based powder. J. Mater. Process. Technol..

[33] Kannatey-Asibu E. (2009). Principles of Laser Materials Processing.

[34] Wilson P.R., Chen Z. (2007). The effect of manganese and chromium on surface oxidation products formed during batch annealing of low carbon steel strip. Corros. Sci..

[35] Saeidi K., Gao X., Zhong Y., Shen Z.J. (2015). Hardened austenite steel with columnar sub-grain structure formed by laser melting. Mater. Sci. Eng..

[36] Kou S. (2003). Welding Metallurgy.

[37] Schuh B., Mendez-Martin F., Völker B., George E.P., Clemens H., Pippan R., Hohenwarter A. (2015). Mechanical properties, microstructure and thermal stability of a nanocrystalline CoCrFeMnNi high-entropy alloy after severe plastic deformation. Acta Mater..

[38] Liu L., Ding Q., Zhong Y., Zou J., Wu J., Chiu Y.-L., Li J., Zhang Z., Yu Q., Shen Z. (2018). Dislocation network in additive manufactured steel breaks strength-ductility trade-off. Mater. Today.

[39] Barkia B., Aubry P., Haghi-Ashtiani P., Auger T., Gosmain L., Schuster F., Maskrot H. (2020). On the origin of the high tensile strength and ductility of additively manufactured 316L stainless steel: Multiscale investigation. J. Mater. Sci. Technol..

